# Time course of changes in inflammatory and oxidative biomarkers in lung tissue of mice induced by exposure to electronic cigarette aerosol

**DOI:** 10.1016/j.toxrep.2022.07.001

**Published:** 2022-08-05

**Authors:** Karem H. Alzoubi, Omar F. Khabour, Nour A. Al-Sawalha, Nareg Karaoghlanian, Alan Shihadeh, Thomas Eissenberg

**Affiliations:** a Department of Clinical Pharmacy, Faculty of Pharmacy, Jordan University of Science and Technology, Irbid, Jordan; b Department of Medical Laboratory Sciences, Faculty of Applied Medical Sciences, Jordan University of Science and Technology, Irbid, Jordan; c Mechanical Engineering Department, American University of Beirut, Beirut 1107 2020, Lebanon; d Department of Psychology, Virginia Commonwealth University, Richmond, VA, USA; e Center for the Study of Tobacco Products, Virginia Commonwealth University, Richmond, VA, USA

**Keywords:** BALF, Bronchoalveolar lavage fluid, e-cigarettes, Electronic cigarette, GPx, Glutathione peroxidase, IL-6, Interleukin-6, IL-10, Interleukin-10, GSSG, Oxidized glutathione, GSH, Reduced glutathione, SOD, Superoxide dismutase, TBARS, Thiobarbituric acid reactive substances, TNFα, Tumor necrosis factor α, E-cigarettes, Aerosol, TNFα, IL-10, IL-6, Lung

## Abstract

**Significance:**

Electronic cigarettes (e-cigarettes) have become a popular way to smoke all over the world. Chronic exposure to e-cigarette aerosol may influence lung health. This study uses an animal model to explore the time course of the effect of exposure to e-cigarette aerosols on the lung.

**Methods:**

Lung samples were collected after exposure of Balb/c mice to e-cigarette aerosols for 1 h/day (6 times/week) for 1, 2 and 4 weeks and compared to sham-exposed controls. Examined biomarkers including inflammatory cells, tumor necrosis factor α (TNFα), interleukin-6 (IL-6), interleukin-10 (IL-10), reduced glutathione (GSH), oxidized glutathione (GSSG), glutathione peroxidase (GPx), catalase, superoxide dismutase (SOD), and Thiobarbituric acid reactive substances (TBARS).

**Results:**

Exposure of animals to e-cigarette aerosols induced significant increases (P < 0.05) in total inflammatory cells, eosinophils, macrophages and TNFα in the lung tissue after 1, 2 and 4 weeks of exposure. Furthermore, level of IL-10 significantly decreased, whereas levels of neutrophils and basophils significantly increased (P < 0.05) after 1 week of exposure. Exposure of animals to e-cigarette aerosol also induced significant decreases (P < 0.05) in the GSH/GSSG ratio, and GPx levels after 2 and 4 weeks of exposures. The activity of catalase was also reduced (P < 0.05) after 4 weeks of exposure. Level of TBARS showed a trend of elevation with time and it reached a significant elevation after 4 weeks (P < 0.01).

**Conclusion:**

Current results indicate that inhalation of unflavored e-cigarette aerosol might be associated with inflammation in lung tissue that worsen as the duration of exposure increases. Further experiments including more time points, histopathology and pulmonary physiology experiments are needed to confirm the current results.

## 1 Introduction

Electronic cigarettes (e-cigarettes) produces aerosols for the users to inhale by heating a liquid that usually contains propylene glycol and glycerin, and almost always contains the stimulant drug nicotine and various flavors [1]. E-cigarettes are popular among adolescents and adults all over the world [2]. Although e-cigarettes contain less toxicants than traditional tobacco products, there is limited information about the time-course of health effects of chronic use of e-cigarettes [3], [4], [5]. Potential harm might arise from a variety of sources, including heating the e-cigarette liquid components, leading to thermal degradation into toxic volatile compounds (formaldehyde, acetaldehyde, and others) [1], [6], [7], [8], [9].

E-cigarette aerosols may cause lung damage [10], heart disease [11], cancer [12], increases in blood pressure [7], and cognitive impairment [13]. At the cellular level, e-cigarette aerosols can cause stress-induced mitochondrial hyperfusion [14]. Using an animal asthma model, e-cigarette aerosols have been shown to induce inflammation response in the respiratory tract [15]. We noted that e-cigarette liquid flavorants and other additives can produce respiratory toxicants when heated in the device [8] and therefore, a detailed description of the puffing conditions and ideally an analytical characterization of the experimental aerosol should be provided. However, the time course of lung damage caused by exposure to e-cigarette aerosols has not yet been investigated. Therefore, the current study aimed to test the effect of e-cigarette aerosols on inflammation and oxidative stress biomarkers in lungs at various time points (one, two, and four weeks of exposure) from a liquid containing no flavorants or additives. Thus, the observed effects would be due solely to aerosolizing the three base ingredients that are common to nearly all e-cigarettes: propylene glycol, vegetable glycerin, and nicotine. Outcomes include inflammatory cells, TNFα, IL-6, IL-10, and oxidative stress/antioxidants biomarkers.

## 2 Methods

### 2.1 Animals

Young (age: 6–8 weeks) male mice (Balb/c) were obtained from the animal unit of Jordan University of Science and Technology (JUST, Irbid, Jordan). The study protocol was approved by the ethics committee of JUST. Animals were kept at a 12:12 light/dark cycle at 24 ± 1°C with free access to water and food. The animals were acclimated for ten days prior to the exposure to e-cigarette aerosols. Animals were grouped (12 in each group) randomly into: controls (exposed to fresh air only), and experimental (exposed to e-cigarette aerosols for 1, 2 or 4 weeks). Aerosol exposure was for 1 h/day by whole body exposure regimen. Biomarkers of inflammation/oxidative stress were examined in the control group at all three time points (4 animals/time point). However, because results from all three time points were very similar, they were all lumped together in as one baseline level for each of the assessed biomarkers.

### 2.2 E-cigarette aerosol exposure

The e-cigarette aerosol whole body exposure system and protocol was based on previous studies where it was successful to examine effects of e-cigarettes exposure in animal models [13], [15], [16], [17], [18]. A 1.8 Ω coil (Kanger-Tech, China) powered at 5.76 W was used to produced e-cigarette aerosols. The liquid mixture was purchased from Sigma Aldrich (USA) and contained propylene glycol: glycerol (3:1), and nicotine (18 mg/ml). Generation of aerosols and exposure of animals were as previously described [16], [18]. The exposure system was programmed to produce one four-second puff every 10 s of the exposure period. The dimensions of the exposure chamber were: 38, 25, and 25 cm that correspond to length, width, and height respectively. Fresh air was supplied to the chamber during exposure The mean concentration of aerosols in the chamber was calculated by measuring the weight change of a filter that was routinely installed in the exhaust port of the chamber. The concentration (mg/m^3^) was computed as (delta filter weight)/ (duration of session x flow rate). The mean concentration of the particulate matter (PM) in the chamber during the exposure sessions was 1487 ± 391 mg/m3. Mice in the control group were exposed to fresh-room air only. Details about the major analytes of the e-cigarette aerosol generated using the current exposure system were previously reported [17]. Aerosol uptake by the animals was verified before conduction of the experiments by measuring plasma cotinine levels after one exposure session. Plasma cotinine levels were measured as previously described [19]. The mean plasma cotinine level ( ± SD) in the exposed animals was 109.12 ± 12.1.x ng/ml (n = 6) versus 9.11 ± 1.2 ng/ml (n = 5) in the air-exposed animals (P < 0.01). The animals that were used to verify the model were not used in the experiments.

### 2.3 Bronchoalveolar lavage fluid (BALF) and lung collection

Animals were euthanized 24 h after the last e-cigarette aerosol exposure by administering 2% of xylazine and 10% ketamine through i.p. injection. The collection of BALF was done as described previously and then centrifuged for 5 min. The supernatant was collected to measure the inflammatory mediators. The pellet was suspended in normal saline and hemacytometer (Hausser Scientific, Horsham, PA) was used to count the total inflammatory cells. A cytospin obtained from Cytotek (Netherlands) was used to perform the differential cell count. Cells were stained using Wright-Giemsa procedure. As well, the lungs were extracted and frozen immediately in liquid nitrogen [20].

### 2.4 Biomarkers measurements

The levels of inflammatory mediators were measured in lung tissues as well as BALF. TNF-α, IL-6 and IL-10 were measured by ELISA kits obtained from eBioscience (San Diego, CA). The activities/levels of GPx, GSH and GSSH were assessed by kits obtained from Sigma-Aldrich. Catalase activity and TBARS level were assessed by kits from Caymanchem (MI, USA). The optical density was measured at wavelengths indicated in the kits using a plate reader (ELx800, Bioteak, Winooski, USA).

### 2.5 Statistical analysis

Statistics was achieved using GraphPad Prism program (La Jolla, CA). Data were expressed as mean ± SEM. One-way ANOVA followed by Tukey’s test was used for analysis of different groups. P < 0.05 was used as a threshold for statistical significance.

## 3 Results

### 3.1 Effect of e-cigarettes on inflammatory cells

**Fig. 1:**
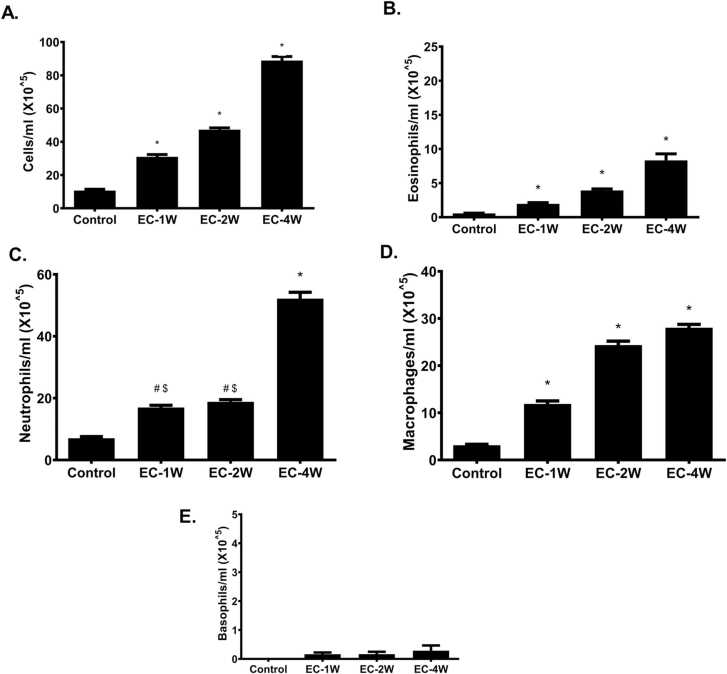
Changes in BALF inflammatory cells after exposure to e-cigarettes. Animals were exposed to e-cigarette aerosols 1 h per day, 6 days per week for 1, 2, and 4 weeks and compared to room air exposed controls. E-cigarettes significantly increased in inflammatory cell counts (A) eosinophils (B) were noticed at all examined time point. In addition, the number of neutrophils (C) and basophils (E) were significantly increased after 1 week of treatment. With respect to macrophages (D), their levels significantly increased after 1 week and 2 weeks of exposure. *indicates a P value of less than 0.05 compared to all other groups, # indicates a P value of less than 0.05 compared to the control group, and $ indicates a P value of less than 0.05 compared to 4 weeks group.

Changes in inflammatory cells were examined in the recovered BALF of mice that were exposed to e-cigarette aerosols for 1 h/day for 1, 2 and 4 weeks compared to controls (Fig. 1 A). E-cigarette aerosols, at all exposure durations, increased the total number of inflammatory cells in BALF of exposed animals (P < 0.05) (Fig. 1 A). In addition, eosinophiles and macrophages counts were significantly increased by e-cigarette aerosol exposure at all exposure durations (P < 0.05) (Figs. 1B, 1D). The number of neutrophils were significantly increased by e-cigarette aerosol exposure after exposure for 1 week (P < 0.05) and 4 weeks of exposure (Fig. 1 C). With respect to basophils, their levels were not affected by e-cigarette aerosol exposure (P > 0.05) (Fig. 1E).

### 3.2 Effect of e-cigarette on inflammatory mediators

**Fig. 2:**
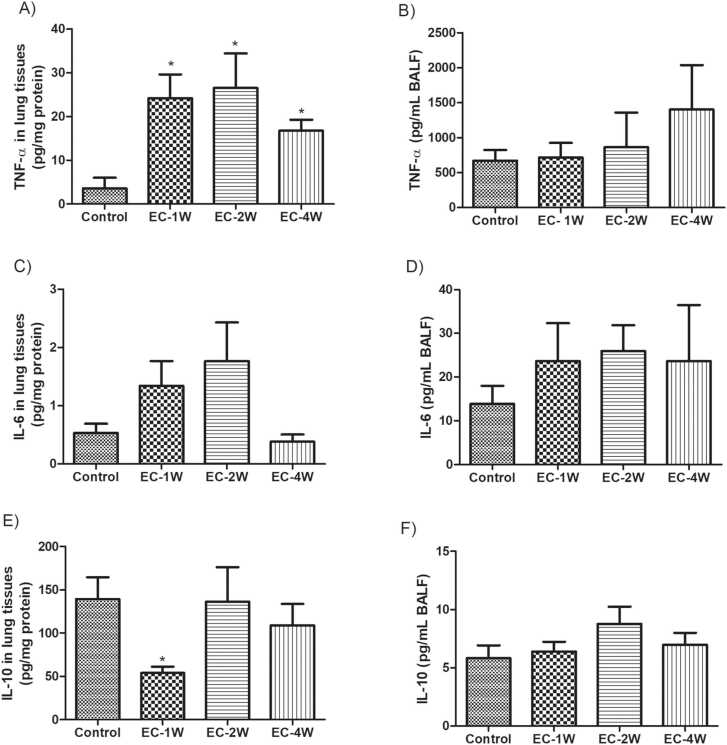
Changes in mediators of inflammation in the lung induced by e-cigarettes. Animals were subjected to e-cigarette aerosols 1 h per day, 6 days per week for 1, 2, and 4 weeks and compared to room air exposed controls. TNF-α in the lungs was significantly increased by e-cigarette aerosol after 1, 2 and 4 weeks of exposure (A), but not in the BALF (B). No significant changes in IL-6 were detected in lung (C) and BALF (D) at all examined time points. IL-10 levels showed significantly decreases in the lung tissue (E) after 1 week of exposure, but its level did not change in the BALF (F). *indicates a P value of less than 0.05.

The levels of TNFα in the lungs were significantly increased by e-cigarette aerosol after 1, 2, and 4 weeks of exposure (P < 0.05) (Fig. 2 A). In BALF preparation, the levels of TNFα were slightly increased with exposure and a notable elevation was observed at 4 weeks of exposure but such elevation was not statistically significant (Fig. 2B). Further, no significant elevation in the level of IL-6 was detected in both lung tissues and BALF preparation by e-cigarette aerosol exposure (Fig. 2 C and D). IL-10 levels in the lung tissue were significantly (P < 0.01) decreased after 1 week of exposure then it adapted to normal levels in the following weeks (Fig. 2E). In the BALF, no significant changes in the level of IL-10 were detected at all examined time points (Fig. 2 F).

### 3.3 Effect of e-cigarette on oxidative stress biomarkers

**Fig. 3:**
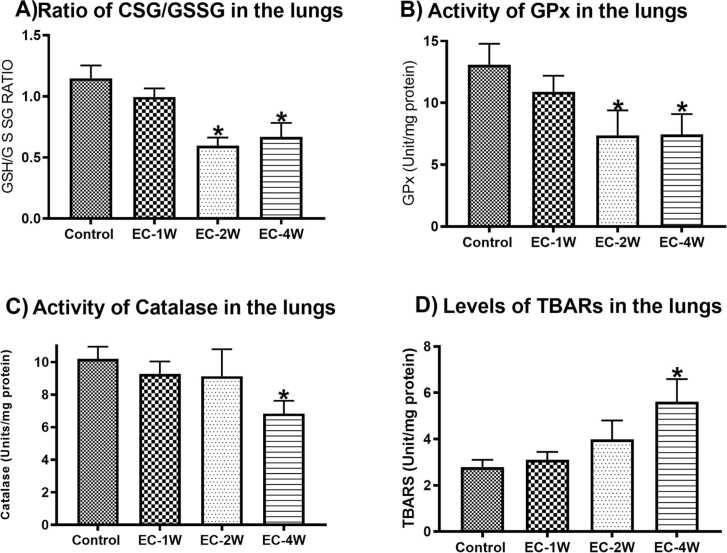
Changes in oxidative stress biomarkers in the lung induced by e-cigarettes. Animals were subjected to e-cigarette aerosols 1 h per day, 6 days per week for 1, 2, and 4 weeks and compared to room air exposed controls. The GSH:GSSG ratio were significantly decreased after 2 and 4 weeks of exposure to e-cigarettes (A). Similarly, GPx was reduced after 2 and 4 weeks of exposure to e-cigarettes (B). With respect to catalase activity, significant decreases were observed after 4 weeks (C). Finally, TBARS levels showed significant elevations after 4 weeks (D). *indicates a P value of less than 0.05.

Exposure to e-cigarette aerosols for 1 week did not affect GSH:GSSG ratio (Fig. 3 A) and TBARS (Fig. 3D). In addition, the activities of GPx (Fig. 3B) and catalase (Fig. 3 C) were not affected compared to control. Two weeks of e-cigarette aerosol exposure significantly (P < 0.05) reduced GSH:GSSG ratio (Fig. 3 A) and the activity of GPx (Fig. 3B) compared to control. However, catalase (Fig. 3 C) and TBARS (Fig. 3D) were not affected (P > 0.05). The longer duration of e-cigarette aerosol exposure, 4 weeks, resulted in significantly (P < 0.05) reduced GSH:GSSG ratio (Fig. 3 A), GPx (Fig. 3B) and catalase (Fig. 3 C). The level of TBARS was significantly elevated in the 4 weeks group (Fig. 3D).

## 4 Discussion

The exposure to e-cigarette aerosols increased the number of recruited inflammatory cells compared to control. Garcia-Arcos and colleagues reported an elevation in inflammatory cells in the lung of mice that were exposed to 2 weeks of nicotine-containing e-cigarette fluids [21]. Further, exposure to e-cigarette aerosol for 3 days, not 4 weeks, increased lung inflammatory cells [22]. This inconsistency in the 4 weeks’ results could be due to the difference in the used mice strain and/or methodological differences.

Different inflammatory cells mediate inflammatory response in the lung after exposure to e-cigarettes. Eosinophils release several inflammatory and cytotoxic mediators that further potentiate airway inflammation and remodeling [23]. E-cigarette aerosols increased eosinophils in the airways compared to unexposed mice. Moreover, the recruited neutrophils play a role in the inflammation and allergic responses that are part of the pathogenic pathway of several airway diseases as asthma, COPD, pulmonary fibrosis, bronchitis and bronchiolitis [24]. The exposure to e-cigarette for 1 week resulted in increased number of recruited neutrophils compared to unexposed animals. This elevation in airway recruitment of neutrophils could be due to reduced levels of IL-10 at this time point. Previous study points toward the role of IL-10 in airway recruitment of neutrophils as tobacco smoke exposure in IL-10 null animals resulted in increased airway recruitment of neutrophils more than the wild type mice [25]. Macrophages and basophils also mediates airway inflammation [26]. The number of macrophages was increased with e-cigarette exposure for 1, 2 and 4 weeks while the basophils were not increased. The result is consistent with previous reports that showed elevated number of retrieved macrophages by exposure to e-cigarettes for 3 days [22] and 4 months [21].

Several inflammatory mediators were altered by e-cigarette aerosol exposure. TNFα is a pro-inflammatory cytokine that activates various types of inflammatory cells [27]. The levels of TNFα in the lungs were increased by e-cigarette aerosol exposure for 1, 2 and 4 weeks. Chen and colleagues showed that long-term e-cigarette exposures to dams before, during and after gestation increased the level of lung’s TNFα [28], [29]. However, a previous study reported exposure to e-cigarettes for 3 days did not affect TNFα levels in the lungs of mice [17]. Similarly, TNFα in the BALF did not change by 3 days or 4 weeks exposure to e-cigarettes [22]. The disagreements in the findings could be due variations in the duration of exposures as well as the used mice strain that affect the airway inflammation in response to allergen [30]. They could as well be due to qualitative differences in the aerosol - e.g., irritants due to intense puffing profiles or coil temperatures.

IL-6 mediates the pathogenesis of asthma and COPD [31]. The present findings showed that e-cigarettes at any time point examined did not affect IL-6 either in lungs or BALF. Consistent result was reported by Glynos and colleagues where IL-6 level was not altered in the BALF of animals that were subjected to either 3 days or 4 weeks of nicotine containing e-cigarette aerosol [22]. IL-10 was originally called as cytokine synthesis inhibitory factor [32]. The present findings showed that e-cigarettes exposure for 1 week transiently decreased IL-10 in the lung while exposure for 2 and 4 weeks did not affect its level. Further studies are needed to examine a wider range of inflammatory mediators as well as anti-inflammatory molecules as IL-13, IL-4, IL-8 among others.

Oxidative stress results when the balance between antioxidants activity and the free radicals is disturbed. The exposure to e-cigarette aerosol generated reactive oxygen species in in vitro studies [33]. The glutathione system is an abundant antioxidant system in the body. The ratio of GSH to GSSG is a primary indicator of the redox balance. The formed GSSG is produced from either the direct scavenging of GSH with radicals or the catalysis of GPx [34]. The current study revealed that the ratio of GSH to GSSG was reduced with e-cigarette aerosol exposure during 2- and 4-weeks’ exposure, pointing toward increased oxidative stress. Further, depletion of intracellular GSH content was observed in cultured human bronchial epithelium cell that were exposed to e-cigarette indicating the presence of oxidative stress [35]. Catalase and GPx are two important antioxidant enzymes that detoxify hydrogen peroxide. The activity of GPx was reduced by e-cigarette aerosols exposure for 2 and 4 weeks and activity of catalase was reduced by exposure for 4 weeks. The reduced activity of GPx by conventional cigarette smoke was revealed in humans and animals [36], [37], [38], [39], [40]. Further, Betsuyaku and colleagues showed that mice that were exposed to repeated cigarette smoke had downregulated catalase in the lungs [41]. Further, chronic nicotine exposure resulted in reduced levels of catalase and GPx in lungs of rats [42]. Further, the current findings reported elevations of TBARS at 4 weeks of e-cigarette exposure. TBARS, as markers of lipid peroxidation, that are formed from reaction of hydrogen peroxide with membrane lipids [43]. This increased lipid peroxidation at 4 weeks could be due to reduced activity of the measured antioxidant enzymes at this time point. However, Glynos and colleagues reported unaltered lung’s and BALF levels of lipid peroxidations, by 3 days and 4 weeks exposure to e-cigarette aerosol [44].

Previous work showed that up to 8 weeks exposure to e-cigarettes vape did not change or marginally increased inflammation in lungs tissues [45], [46]. The current study investigated the time course of oxidative stress and inflammatory changes in the lungs of mice exposed to unflavored e-cigarettes in a subchronic/subacute inhalation study. This study has some limitations. Chronic exposure duration of more than 6 months was not part of this study design. This could a matter of future work. As well, the use of a reference standard, e.g., cigarette smoke, and additional histological analysis would have been ideal. However, for the purposes of the current study, which was focused on lung inflammation and oxidative stress, the used design relaying on cell counts, and biomarker analysis is sufficient. The oxidative stress parameters (GSH/GSSG ratio, GPx activity, catalase activity, TBARs level) gave a consistent picture of decreased GSH/GSSG ratio and GPx activity after 2 and 4 weeks of exposure, followed by decreased catalase activity and increased TBARS levels at 4 weeks, indicating an attenuation of the protection against oxidative stress. The lung inflammation results are less consistent. Future work is warranted to explore larger panels of inflammatory mediators that are more representative for respiratory effects of inhaled e-cigarette aerosols.

The e-cigarettes exposure protocol used in the current study was based on several previous studies that showed its effectiveness in mimicking exposure to e-cigarettes [13], [15], [16], [17], [18]. Levels of major analytes the exposure aerosol were previously reported using this protocol [17]. The puffing intensity of the protocol used in the current study is more intense than other routine recommended regimens for e-cigarette testing such as the Coresta Method 81 [47]. However, even these standard regimens may not truly represent human puffing behavior, and human topography measurements may provide better representation [48]. The study examined changes induced by e-cigarettes at only three time points (one week, two weeks, and four weeks). Therefore, it is highly recommended to expand the study to include shorter (1 day) and longer (months) exposure periods. In addition, the collection of histopathological and respiratory data could provide further insight into the effects of e-cigarettes on the lung.

In summary, the results showed that the exposure to unflavored e-cigarette aerosol for different durations increased inflammatory cells in the lungs. In addition, e-cigarette aerosols exposure altered the level of inflammatory mediators that further potentiate immune response in the lung. Further, e-cigarette is associated with lung oxidative stress where the activities of several antioxidant enzymes were reduced, and the lipid peroxidation byproduct was induced. These findings point toward the harmful health effects of e-cigarettes on the lungs, even when no flavorants or other additives were present. This study demonstrates that e-cigarette emissions may be intrinsically hazardous to the lung. Further research is warranted to examine the different molecules that could mediate the airway inflammation seen in the current study. This includes more time points, histopathology and pulmonary physiology experiments are needed to confirm the current results.

## Funding

This study was supported by a grant (no. 368/2015) from the Deanship of Research at 10.13039/501100004035Jordan University of Science and Technology, Irbid, Jordan. Drs. Eissenberg and Shihadeh are supported by grant number U54DA036105 from the National Institute on Drug Abuse of the National Institutes of Health and the Center for Tobacco Products of the U.S. Food and Drug Administration. The content is solely the responsibility of the authors and does not necessarily represent the views of the NIH or the FDA.

## CRediT authorship contribution statement

**Karem H. Alzoubi**: Conceptualization, Data curation, Formal analysis, Funding acquisition, Investigation, Methodology, Project administration, Resources, Software, Supervision, Validation, Visualization, Writing – original draft, Writing – review & editing. **Omar F. Khabour**: Conceptualization, Data curation, Formal analysis, Funding acquisition, Investigation, Methodology, Project administration, Resources, Software, Supervision, Writing – original draft, Writing – review & editing. **Nour Al-Sawalha**: Conceptualization, Data curation, Formal analysis, Investigation, Methodology, Project administration, Resources, Software, Supervision, Writing – original draft, Writing – review & editing. **Nareg Karaoghlanian**: Investigation, Methodology, Resources, Software, Supervision, Validation, Visualization, Review & editing of manuscript. **Alan Shihadeh**: Conceptualization, Funding acquisition, Investigation, Methodology, Project administration, Resources, Software, Review & editing of the manuscript. **Thomas Eissenberg**: Conceptualization, Funding acquisition, Project administration, Resources, Software, Supervision, Review & editing of the manuscript.

## Declaration of Competing Interest

The authors declare the following financial interests/personal relationships which may be considered as potential competing interests: Drs. Eissenberg and Shihadeh are paid consultants in litigation against the tobacco industry and also the electronic cigarette industry and are named on one patent for a device that measures the puffing behavior of electronic cigarette users. Dr. Eissenberg is also named on another patent application for a smartphone app that determines electronic cigarette device and liquid characteristics, and a third patent application for a smoking cessation intervention. Other authors have no conflict of interest.

## References

[1] Korfei M. (2018). The underestimated danger of E-cigarettes - also in the absence of nicotine. Respir. Res..

[2] Breland A., Soule E., Lopez A., Ramôa C., El-Hellani A., Eissenberg T. (2017). Electronic cigarettes: what are they and what do they do?. Ann. N. Y. Acad. Sci..

[3] Sifat A.E., Vaidya B., Kaisar M.A., Cucullo L., Abbruscato T.J. (2018). Nicotine and electronic cigarette (E-Cig) exposure decreases brain glucose utilization in ischemic stroke. J. Neurochem..

[4] Perikleous E.P., Steiropoulos P., Paraskakis E., Constantinidis T.C., Nena E. (2018). E-cigarette use among adolescents: an overview of the literature and future perspectives. Front. Public Health.

[5] Eissenberg T., Bhatnagar A., Chapman S., Jordt S.-E., Shihadeh A., Soule E.K. (2020). Invalidity of an oft-cited estimate of the relative harms of electronic cigarettes. Am. J. Public Health.

[6] DeVito E.E., Krishnan-Sarin S. (2018). E-cigarettes: impact of E-liquid components and device characteristics on nicotine exposure. Curr. Neuropharmacol..

[7] Qasim H., Karim Z., Rivera J.O., Khasawneh F.T., Alshbool F.Z. (2017). Impact of electronic cigarettes in the cardiovascular system. J. Am. Heart Assoc..

[8] El-Hellani A., El-Hage R., Salman R., Talih S., Zeaiter J., Eissenberg T., Shihadeh A., Saliba N.A. (2020). Electronic cigarettes are chemical reactors: implication to toxicity. Chem. Res. Toxicol..

[9] Talih S., Salman R., Karam E., El-Hourani M., El-Hage R., Karaoghlanian N., El-Hellani A., Saliba N., Shihadeh A. (2020). Hot wires and film boiling: another look at carbonyl formation in electronic cigarettes. Chem. Res. Toxicol..

[10] Madison M.C., Landers C.T., Gu B.-H., Chang C.-Y., Tung H.-Y., You R., Hong M.J., Baghaei N., Song L.-Z., Porter P., Putluri N., Salas R., Gilbert B.E., Levental I., Campen M.J., Corry D.B., Kheradmand F. (2019). Electronic cigarettes disrupt lung lipid homeostasis and innate immunity independent of nicotine. J. Clin. Investig..

[11] Ogunwale M.A., Li M., Ramakrishnam Raju M.V., Chen Y., Nantz M.H., Conklin D.J., Fu X.-A. (2017). Aldehyde detection in electronic cigarette aerosols. ACS Omega.

[12] Rowell T.R., Tarran R. (2015). Will chronic e-cigarette use cause lung disease?. Am. J. Physiol. Lung Cell Mol. Physiol..

[13] Alzoubi K.H., Batran R.M., Al-Sawalha N.A., Khabour O.F., Karaoghlanian N., Shihadeh A., Eissenberg T. (2021). The effect of electronic cigarettes exposure on learning and memory functions: behavioral and molecular analysis. Inhal. Toxicol..

[14] Zahedi A., Phandthong R., Chaili A., Leung S., Omaiye E., Talbot P. (2019). Mitochondrial stress response in neural stem cells exposed to electronic cigarettes. iScience.

[15] Taha H.R., Al-Sawalha N.A., Alzoubi K.H., Khabour O.F. (2020). Effect of E-Cigarette aerosol exposure on airway inflammation in a murine model of asthma. Inhal. Toxicol..

[16] Al-Sawalha N., Alzoubi K., Khabour O., Karaoghlanian N., Ismail Z., Shihadeh A., Eissenberg T. (2020). Effect of electronic cigarette aerosol exposure during gestation and lactation on learning and memory of adult male offspring rats. Physiol. Behav..

[17] Husari A., Shihadeh A., Talih S., Hashem Y., El Sabban M., Zaatari G. (2016). Acute exposure to electronic and combustible cigarette aerosols: effects in an animal model and in human alveolar cells. Nicotine Tob. Res..

[18] Mayyas F., Aldawod H., Alzoubi K.H., Khabour O., Shihadeh A., Eissenberg T. (2020). Comparison of the cardiac effects of electronic cigarette aerosol exposure with waterpipe and combustible cigarette smoke exposure in rats. Life Sci..

[19] Massadeh A.M., Gharaibeh A.A., Omari K.W. (2009). A single-step extraction method for the determination of nicotine and cotinine in Jordanian smokers’ blood and urine samples by RP-HPLC and GC-MS. J. Chromatogr. Sci..

[20] Al-Sawalha N.A., Migdadi A.M., Alzoubi K.H., Khabour O.F., Qinna N.A. (2017). Effect of waterpipe tobacco smoking on airway inflammation in murine model of asthma. Inhal. Toxicol..

[21] Garcia-Arcos I., Geraghty P., Baumlin N., Campos M., Dabo A.J., Jundi B., Cummins N., Eden E., Grosche A., Salathe M., Foronjy R. (2016). Chronic electronic cigarette exposure in mice induces features of COPD in a nicotine-dependent manner. Thorax.

[22] Glynos C., Bibli S.I., Katsaounou P., Pavlidou A., Magkou C., Karavana V., Topouzis S., Kalomenidis I., Zakynthinos S., Papapetropoulos A. (2018). Comparison of the effects of e-cigarette vapor with cigarette smoke on lung function and inflammation in mice.

[23] George L., Brightling C.E. (2016). Eosinophilic airway inflammation: role in asthma and chronic obstructive pulmonary disease. Ther. Adv. Chronic Dis..

[24] Liu J., Pang Z., Wang G., Guan X., Fang K., Wang Z., Wang F. (2017). Advanced role of neutrophils in common respiratory diseases.

[25] Higaki M., Wada H., Mikura S., Yasutake T., Nakamura M., Niikura M., Kobayashi F., Kamma H., Kamiya S., Ito K., Barnes P.J., Goto H., Takizawa H. (2015). Interleukin-10 modulates pulmonary neutrophilic inflammation induced by cigarette smoke exposure. Exp. Lung Res..

[26] Kay A.B. (1986). The cells causing airway inflammation. Eur. J. Respir. Dis..

[27] Russo C., Polosa R. (2005). TNF-alpha as a promising therapeutic target in chronic asthma: a lesson from rheumatoid arthritis. Clin. Sci..

[28] American journal of respiratory cell and molecular biology.

[29] Chen H., Li G., Chan Y.L., Chapman D.G., Sukjamnong S., Nguyen T., Annissa T., McGrath K.C., Sharma P., Oliver B.G. (2018). Maternal E-Cigarette Exposure in Mice Alters DNA Methylation and Lung Cytokine Expression in Offspring.

[30] Kelada S.N., Wilson M.S., Tavarez U., Kubalanza K., Borate B., Whitehead G.S., Maruoka S., Roy M.G., Olive M., Carpenter D.E., Brass D.M., Wynn T.A., Cook D.N., Evans C.M., Schwartz D.A., Collins F.S. (2011). Strain-dependent genomic factors affect allergen-induced airway hyperresponsiveness in mice. Am. J. Respir. Cell Mol. Biol..

[31] Rincon M., Irvin C.G. (2012). Role of IL-6 in asthma and other inflammatory pulmonary diseases. Int. J. Biol. Sci..

[32] Ogawa Y., Duru E.A., Ameredes B.T. (2008). Role of IL-10 in the resolution of airway inflammation. Curr. Mol. Med..

[33] Zhao J., Zhang Y., Sisler J.D., Shaffer J., Leonard S.S., Morris A.M., Qian Y., Bello D., Demokritou P. (2018). Assessment of reactive oxygen species generated by electronic cigarettes using acellular and cellular approaches. J. Hazard. Mater..

[34] Aquilano K., Baldelli S., Ciriolo M.R. (2014). Glutathione: new roles in redox signaling for an old antioxidant. Front. Pharmacol..

[35] Czekala L., Simms L., Stevenson M., Trelles-Sticken E., Walker P., Walele T. (2019). High Content Screening in NHBE cells shows significantly reduced biological activity of flavoured e-liquids, when compared to cigarette smoke condensate. Toxicol. In Vitro.

[36] Valenca S.S., Silva Bezerra F., Lopes A.A., Romana-Souza B., Marinho Cavalcante M.C., Lima A.B., Goncalves Koatz V.L., Porto L.C. (2008). Oxidative stress in mouse plasma and lungs induced by cigarette smoke and lipopolysaccharide. Environ. Res..

[37] Zhou J.F., Yan X.F., Guo F.Z., Sun N.Y., Qian Z.J., Ding D.Y. (2000). Effects of cigarette smoking and smoking cessation on plasma constituents and enzyme activities related to oxidative stress. Biomed. Environ. Sci..

[38] Kondo T., Tagami S., Yoshioka A., Nishimura M., Kawakami Y. (1994). Current smoking of elderly men reduces antioxidants in alveolar macrophages. Am. J. Respir. Crit. Care Med..

[39] Codandabany U. (2000). Erythrocyte lipid peroxidation and antioxidants in cigarette smokers. Cell Biochem. Funct..

[40] Rahman I., MacNee W. (1996). Role of oxidants/antioxidants in smoking-induced lung diseases. Free Radic. Biol. Med..

[41] Betsuyaku T., Fuke S., Inomata T., Kaga K., Morikawa T., Odajima N., Adair-Kirk T., Nishimura M. (2013). Bronchiolar epithelial catalase is diminished in smokers with mild COPD. Eur. Respir. J..

[42] Kalpana C., Menon V.P. (2004). Curcumin ameliorates oxidative stress during nicotine-induced lung toxicity in Wistar rats. Ital. J. Biochem..

[43] Antczak A., Nowak D., Shariati B., Krol M., Piasecka G., Kurmanowska Z. (1997). Increased hydrogen peroxide and thiobarbituric acid-reactive products in expired breath condensate of asthmatic patients. Eur. Respir. J..

[44] Glynos C., Bibli S.I., Katsaounou P., Pavlidou A., Magkou C., Karavana V., Topouzis S., Kalomenidis I., Zakynthinos S., Papapetropoulos A. (2018). Comparison of the effects of e-cigarette vapor with cigarette smoke on lung function and inflammation in mice. Am. J. Physiol. Lung Cell Mol. Physiol..

[45] Szafran B.N., Pinkston R., Perveen Z., Ross M.K., Morgan T., Paulsen D.B., Penn A.L., Kaplan B.L.F., Noël A. (2020). Electronic-cigarette vehicles and flavoring affect lung function and immune responses in a murine model. Int. J. Mol. Sci..

[46] Larcombe A.N., Janka M.A., Mullins B.J., Berry L.J., Bredin A., Franklin P.J. (2017). The effects of electronic cigarette aerosol exposure on inflammation and lung function in mice. Am. J. Physiol. Lung Cell Mol. Physiol..

[47] CORESTA (2015).

[48] Kosmider L., Kimber C.F., Kurek J., Corcoran O., Dawkins L.E. (2018). Compensatory puffing with lower nicotine concentration e-liquids increases carbonyl exposure in e-cigarette aerosols. Nicotine Tob. Res..

